# The Role of Platelet Rich Plasma in Vertebrogenic and Discogenic Pain: A Systematic Review and Meta-Analysis

**DOI:** 10.1007/s11916-024-01274-y

**Published:** 2024-06-08

**Authors:** Saurabh Kataria, Jeremiah Hilkiah Wijaya, Utsav Patel, Kevin Yabut, Tawfiq Turjman, Muhammad Abubakar Ayub, Nihar Upadhyay, Moinulhaq Makrani, Hisham Turjman, Ahmed Mostafa Abdalla Mohamed, Alan D. Kaye

**Affiliations:** 1 Department of Neurology, Louisiana State University Health Sciences Center at Shreveport, 1501 Kings Highway, Shreveport, LA 71103 USA; 2 Department of Neurosurgery, Siloam Hospital Lippo Village, Tangerang, Banten, Indonesia; 3 Mayo Clinic, Jacksonville, FL 32224 USA; 4 Louisiana State University Health Sciences Center, Shreveport, LA 71103 USA; 5 School of Medicine, Royal College of Surgeons in Ireland, Baharain, USA; 6 Department of Internal Medicine, GMERS Medical College, Gotri, Vadodara, Gujarat 390021 India; 7 Dept. of Pharmacology, Parul Institute of Medical Science and Research, Waghodiya, Gujarat, 291760 India; 8 Department of Anaesthesiology and Interventional Pain, Louisiana State University Health Sciences Center at Shreveport, Shreveport, LA 71103 USA; 9 Louisiana Addiction Research Center, Shreveport, LA 71103 USA

**Keywords:** Back pain, Low back pain, Vertebrogenic pain, Discogenic pain, Platelet-rich plasma

## Abstract

**Purpose of Review:**

The present investigation evaluates clinical uses and roles of platelet rich plasma in the management of vetrebrogenic and discogenic mediated pain states.

**Recent Findings:**

Back pain is a common and significant condition that affects millions of people around the world. The cause of back pain is often complex and multifactorial, with discogenic and vertebrogenic pain being two subtypes of back pain. Currently, there are numerous methods and modalities in which back pain is managed and treated such as physical therapy, electrical nerve stimulation, pharmacotherapies, and platelet-rich plasma. To conduct this systematic review, the authors used the keywords “platelet-rich plasma”, “vertebrogenic pain”, and “discogenic pain”, on PubMed, EuroPMC, Who ICTRP, and clinicaltrials.gov to better elucidate the role of this treatment method for combating vertebrogenic and discogenic back pain. In recent decades, there has been a rise in popularity of the use of platelet-rich plasma for the treatment of numerous musculoskeletal conditions. Related to high concentration of platelets, growth factors, cytokines, and chemokines, platelet-rich plasma is effective in reducing pain related symptoms and in the treatment of back pain.

**Summary:**

Platelet-rich plasma use has evolved and gained popularity for pain related conditions, including vertebrogenic and discogenic back pain. Additional well-designed studies are warranted in the future to better determine best practice strategies to provide future clinicians with a solid foundation of evidence to make advancements with regenerative medical therapies such as platelet-rich plasma.

**Supplementary Information:**

The online version contains supplementary material available at 10.1007/s11916-024-01274-y.

## Introduction

The management of discogenic and vertebrogenic pain has encountered both advancements and challenges. In this regard, clinical research has delved into application of platelet-rich plasma (PRP) as a potential remedy for these complex ailments. An initial clinical trial was conducted to assess safety and initial effectiveness of intradiscal injection of autologous PRP for discogenic low back pain. The trial exhibited promising outcomes, suggesting the viability of PRP as a potentially effective treatment.

Additionally, optimistic preliminary results were disclosed from a prospective trial utilizing intradiscal PRP for chronic discogenic lower back pain. These findings lend support to PRP as a potential treatment option for vertebral disc-related pain. Further, an examination of prolonged effects of intradiscal PRP injections for moderate-to-severe lumbar discogenic pain revealed noteworthy enhancements in pain relief and functional improvement at 5 to 9 years post-injection. This implies the potential of sustained efficacy with PRP in addressing discogenic pain. Moreover, another study advocated for utilization of intradiscal PRP in treating discogenic pain, underscoring the significance of higher platelet counts for a positive response.

Despite promising evidence supporting use of PRP for discogenic pain, large-scale studies are limited regarding clinical efficacy. Hence, we aimed to assess in the present investigation safety and efficacy of PRP in treating patients with discogenic and vertebrogenic pain.

## Methods

The present investigation involved a systematic review. A set of MeSH-based search queries were generated to explore research on utilization of PRP in management of vertebrogenic and discogenic pain (see Table 1). These queries encompass a range of relevant terms and concepts, aiding in the comprehensive retrieval of pertinent literature from medical databases such as PubMed, EuroPMC, WHO ICTRP, and Clinicaltrials.gov. We utilized the following search terms to systematically review available evidence on this topic: "Platelet-rich plasma," "Vertebrogenic Pain," and "Discogenic Pain", facilitating a thorough investigation into potential benefits and outcomes associated with PRP therapy for individuals suffering from vertebrogenic or discogenic pain. Additionally, we performed manual searching as part of our systematic review process.

**Table 1:** Detailed search strategy use for PubMed and EuroPMC to retrieve paper discussing the use of PRP in vertebrogenic and discogenic pain

**Search source**	**Search queries**
PubMed	("Intervertebral Disc Displacement"[All Fields] OR "Intervertebral Disc Degeneration"[All Fields] OR "Spinal Diseases"[All Fields] OR "back pain"[All Fields] OR "axial pain"[All Fields]) AND ("Spinal pain"[All Fields] OR "Vertebral pain"[All Fields] OR "back pain"[All Fields] OR "Spine-related pain"[All Fields] OR "Vertebral column pain"[All Fields] OR "axial pain"[All Fields] OR "Radicular pain"[All Fields] OR "Discogenic pain"[All Fields] OR "Spondylogenic pain"[All Fields]) AND ("Platelet-Rich Plasma"[All Fields] OR "Platelet Concentrates"[All Fields] OR "Autologous Blood Transfusion"[All Fields] OR "Growth Factors"[All Fields] OR "Blood Platelets"[All Fields])
EuroPMC	"Spinal pain" OR "Vertebral pain" OR "Back pain" OR "Spine-related pain" OR "Vertebral column pain" OR "Axial pain" OR "Radicular pain" OR "Discogenic pain" OR "Spondylogenic pain" AND "Platelet-Rich Plasma" OR "Platelet Concentrates" OR "Autologous Blood Transfusion" OR "Growth Factors" OR "Blood Platelets"
Clinicaltrials.gov	"Spinal pain" OR "Vertebral pain" OR "Back pain" OR "Spine-related pain" OR "Vertebral column pain" OR "Axial pain" OR "Radicular pain" OR "Discogenic pain" OR "Spondylogenic pain" AND "Platelet-Rich Plasma" OR "Platelet Concentrates" OR "Autologous Blood Transfusion" OR "Growth Factors" OR "Blood Platelets"
WHO ICTRP	"Spinal pain" OR "Vertebral pain" OR "Back pain" OR "Spine-related pain" OR "Vertebral column pain" OR "Axial pain" OR "Radicular pain" OR "Discogenic pain" OR "Spondylogenic pain" AND "Platelet-Rich Plasma" OR "Platelet Concentrates" OR "Autologous Blood Transfusion" OR "Growth Factors" OR "Blood Platelets"

In terms of inclusion criteria, we included randomized controlled trials (RCTs) that focus on PRP as a treatment for vertebrogenic or discogenic pain in individuals of greater than 18 years of age. These studies compared PRP treatment with standard care, placebo, or alternative interventions and report outcomes related to pain reduction, functional improvement, quality of life, adverse events, or radiological assessments. Excluded from our analysis were case reports, case series, animal studies, letters, editorials, and conference abstracts. Studies not related to vertebrogenic or discogenic pain, those involving regenerative therapies other than PRP, lacking relevant comparators, or not reporting relevant outcome data were excluded. Additionally, studies published in languages other than English were excluded, unless translation resources were adequate.

In this systematic review, the process of determining whether a study met the inclusion criteria of the review was conducted with a structured and rigorous approach. All authors were responsible for screening each record and report retrieved, working independently of each other to minimize bias. Automation tools were not utilized in this process; instead, the reviewers manually assessed each record and report to ensure a comprehensive and thorough evaluation. In cases where discrepancies or disagreements arose between reviewers regarding the inclusion or exclusion of a particular study, a consensus meeting was convened to resolve differences through discussion and mutual agreement. If a consensus could not be reached, a senior reviewer was consulted to make the ultimate determination.

The primary outcomes included pain assessment, as measured through instruments such as the numerical rating scale (NRS), visual analogue score (VAS) and numerical pain scale (NPS), as well as disability evaluation employing the Oswestry Disability Index (ODI). These evaluations encompassed the determination of both absolute scores and alterations from baseline scores at various time intervals, including baseline and follow-up periods post-PRP injection. Measures of effect was derived in two distinct approaches. First, the average absolute value for each time point was computed, entailing calculation of mean pain or disability scores across the entire patient cohort at a specific time interval. Secondly, mean difference for each time point concerning baseline values was determined, signifying change in pain or disability scores relative to values recorded prior to cell- or PRP injection.

The data extraction process for this study followed a methodical approach. Initially, potential articles were subjected to a two-stage screening process. Data extraction was carried out using a standardized sheet, with all reviewers independently recording evaluations of study design, patient characteristics, and treatment particulars. Moreover, various outcome measures, such as VAS, NPS, or NRS, ODI disability scores, or other scoring system. Additionally, quantitative data pertaining to quality of life, radiographic outcomes, and adverse event was extracted.

The Cochrane Risk of Bias (ROB) tool, developed by the Cochrane Collaboration, is a methodological framework used to assess internal validity of studies, particularly randomized controlled trials (RCTs), included in systematic reviews and meta-analyses by three independent authors (JH, UD, and SK). It evaluates various domains, including random sequence generation, allocation concealment, blinding of participants and personnel, blinding of outcome assessment, incomplete outcome data, selective reporting, and other potential sources of bias. Each domain is rated as "unclear", "low," or "high" risk of bias based on available information, and an overall risk of bias for a study is determined through collective assessments. The Cochrane ROB tool aids in transparently gauging the reliability and trustworthiness of evidence, facilitating informed judgments about the quality of studies incorporated into current systematic reviews and meta-analyses.

To conduct a meta-analysis, Review Manager 5.3 (Copenhagen: The Cochrane Collaboration, 2014) was employed. Continuous variables were expressed as means accompanied by mean standard deviations (MSDs), computed using the inverse-variance methodology. Utilizing random effects models regardless of heterogeneity, mean standard differences (MSDs) for continuous variables were presented with confidence intervals (CIs) of 95%. Employing a two-tailed P-value, statistical significance was established at ≤ 0.05. Assessment of heterogeneity involved the Q-statistic test and the I2 test. The I2 statistic quantified the proportion of overall variability stemming from clinical or methodological heterogeneity as opposed to chance. Significance (P < 0.05) in the Q statistics denoted heterogeneity among the studies, with I2 values exceeding 50% indicating substantial heterogeneity.

## Results

**Fig. 1:**
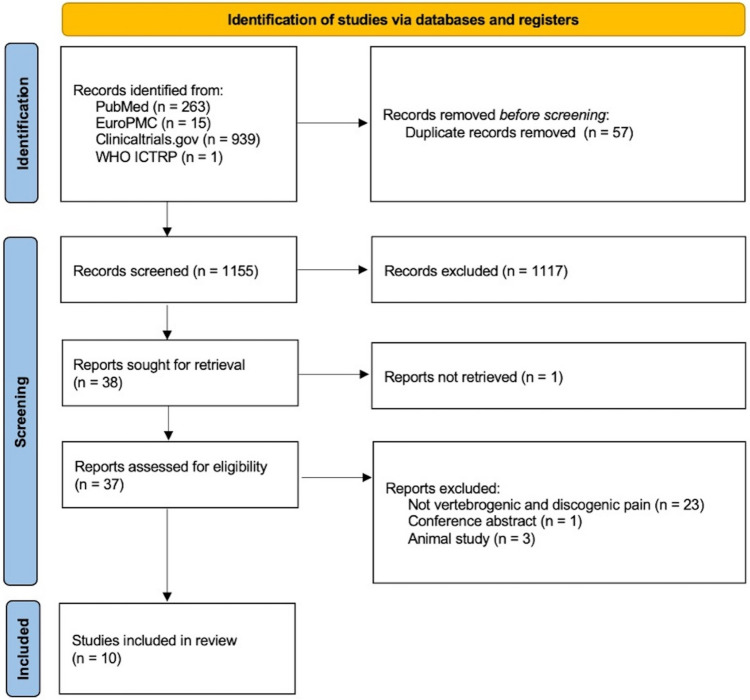
Flow diagram following the PRISMA guidelines illustrating the process of selecting studies for inclusion [1, 2•, 3•, 4, 5•, 6, 7•, 8•, 9•, 10, 11]

The records considered for inclusion in the present investigation were derived from diverse sources, namely PubMed (n = 263), EuroPMC (n = 15), Clinicaltrials.gov (n = 939), and WHO ICTRP (n = 1). Prior to the screening process, a culling of 57 duplicate records was executed. In the subsequent phase of title and abstract screening, a comprehensive evaluation of 1155 records transpired, leading to exclusion of 1117 records. The retrieval process sought 38 reports, with only one remaining unretrieved. Following this, 37 reports underwent eligibility assessment. After an exhaustive full-text screening, numerous reports were excluded based on specific criteria: 23 related to lack of relevance to vertebrogenic and to discogenic pain, 1 because it was a conference abstract, and 3 because they constituted animal studies. In totality, the systematic review incorporates a compilation of 10 studies (see Fig. 1 PRISMA) [1, 2•, 3•, 4, 5•, 6, 7•, 8•, 9•, 10, 11].

As shown in Table 2 below, it consists of a comparative synthesis of diverse clinical studies concentrating on the management of pain. These studies encompass a spectrum of conditions such as lumbar facet joint syndrome to chronic non-specific low back pain. The interventions predominantly involve PRP administered in different quantities and combinations, juxtaposed against various control substances. The studies exhibit varying follow-up durations ranging from 3 to 12 months, facilitating the evaluation of both short-term and long-term treatment effects.

**Table 2:** Comparative synthesis of regenerative medicine clinical studies concentrating on PRP in the management of musculoskeletal pain

**Study ID**	**Type of pain**	**Total cohort, n**	**Age, years**	**Male, n**	**Intervention details**	**Control details**	**Route of administration**	**Follow up duration**
Wu (2017) [1]	Lumbar facet joint syndrome	23 vs 23	52.91 ± 7.60 vs 52.78 ± 7.25	10 vs 9	0.5 mL of autologous PRP for every targeted joint	0.5% lidocaine and 5 mg/mL betamethasone	Intra-articular	6 months
Tuakli-Wosornu et al. [2•]	Chronic lumbar discogenic pain	29 vs 18	41.40 ± 8.08 vs 43.80 ± 8.91	14 vs 2	3 – 4 mL of PRP	Contrast agent	Intradiscal	12 months
Singla et al. [3•]	Sacroiliac joint pain	20 vs 20	35.20 ± 12.86 vs 37.00 ± 10.89	16 vs 16	3 mL of PRP with 0.5 mL of calcium chloride	1.5 g of methylprednisolone and 1.5 mL of 2% lidocaine with 0.5 mL 0.9% sterile saline	Sacroiliac joint	3 months
Xu et al. [4]	Lumbar disc herniation	61 vs 63	56.0 (44.5–60.0) vs 56.0 (50.0–59.0)	28 vs 37	3 mL of PRP	2 ml betamethasone + 0.5 ml 0.9% sterile saline + 0.5 ml 2% lidocaine	Transforaminal	12 months
Won et al. [5•]	Chronic non-specific low back pain	14 vs 16	51.0 ± 18.1 vs 50.5 ± 17.0	6 vs 6	5 – 6 mL of PRP, intradiscal	6 mL 0.5% lidocaine	Ligament and muscular	6 months
Ruiz-Lopez and Tsai [6]	Chronic degenerative spinal disease	25 vs 25	68 ± 13.06 vs 61 ± 12.60	11 vs 10	16.5 mL of PRP with 3.5 mL 0.9% sterile saline	60 mg of triamcinolone acetonide and 3.5 0.9% sterile saline	Epidural	6 months
Akeda (2023) [16•]	Discogenic low back pain	44 vs 45	40.3 ± 10.4 vs 39.1 ± 11.5	16 vs 19	1 mL of PRP	1 mL 0.9% sterile saline and 0.2 g Kefzol	Intradiscal	12 months
Zielinski et al. [9•]	Lumbar discogenic pain	18 vs 8	n/r	n/r	2 mL of PRP	3 mL 0.9% sterile saline	Intradiscal	8 weeks
Mohi Eldin et al. [10]	Discogenic low back pain	44 vs 88	< 40 years: 63 ≥ 40 years: 69	n/r	3 mL of PRP	3 mL of PRF	Intradiscal	6 months
Akeda (2022) [7•]	Discogenic low back pain	9 vs 7	35.1 ± 8.7 vs 27.9 ± 5.2	6 vs 5	2 mL of PRP	2 mg betamethasone and 2 mL 0.9% sterile saline	Intradiscal	12 months

Data in “vs” was displayed as intervention vs control

Our meta-analysis (see Table 3) on VAS and ODI scores at different intervals. For VAS, Supplementary Fig. 1 shows that in the first month, derived from 7 studies, the Standardized Mean Difference (SMD) was -0.14 (95% CI of -0.62 to 0.34, p = 0.57), indicating no statistically significant difference between the PRP and control groups. Similarly as per Supplementary Figs. 2 and 3, at the third month (SMD: -0.27, 95% CI: -0.85 to 0.31, p = 0.36) and the sixth month (SMD: -0.14, 95% CI: -0.99 to 0.72, p = 0.75), there were no statistically significant distinctions.

**Table 3:** Summary of meta-analysis

**Variables**	**# studies**	**Participants**	**Std. mean difference (IV, Random, 95% CI)**	**p-value**
**VAS**				
VAS 1st month	7	307	-0.14 [-0.62, 0.34]	0.57
VAS 3rd month	6	392	-0.27 [-0.85, 0.31]	0.36
VAS 6th month	5	382	-0.14 [-0.99, 0.72]	0.75
**ODI**				
ODI 1st month	3	170	0.29 [-0.01, 0.59]	0.06
ODI 3rd month	3	170	-0.03 [-0.50, 0.43]	0.89
ODI 6th month	2	154	-0.14 [-0.46, 0.18]	0.39

In comparison For ODI scores, as depicted in Supplementary Fig. 4; in the first month, based on data from 3 studies, the SMD was 0.29 (95% CI: -0.01 to 0.59, p = 0.06), indicating a trend towards significance. However, Supplementary Figs. 5 and 6; at the third month (SMD: -0.03, 95% CI: -0.50 to 0.43, p = 0.89) and the sixth month (SMD: -0.14, 95% CI: -0.46 to 0.18, p = 0.39), shows no statistically significant differences were noticed.

**Fig. 2:**
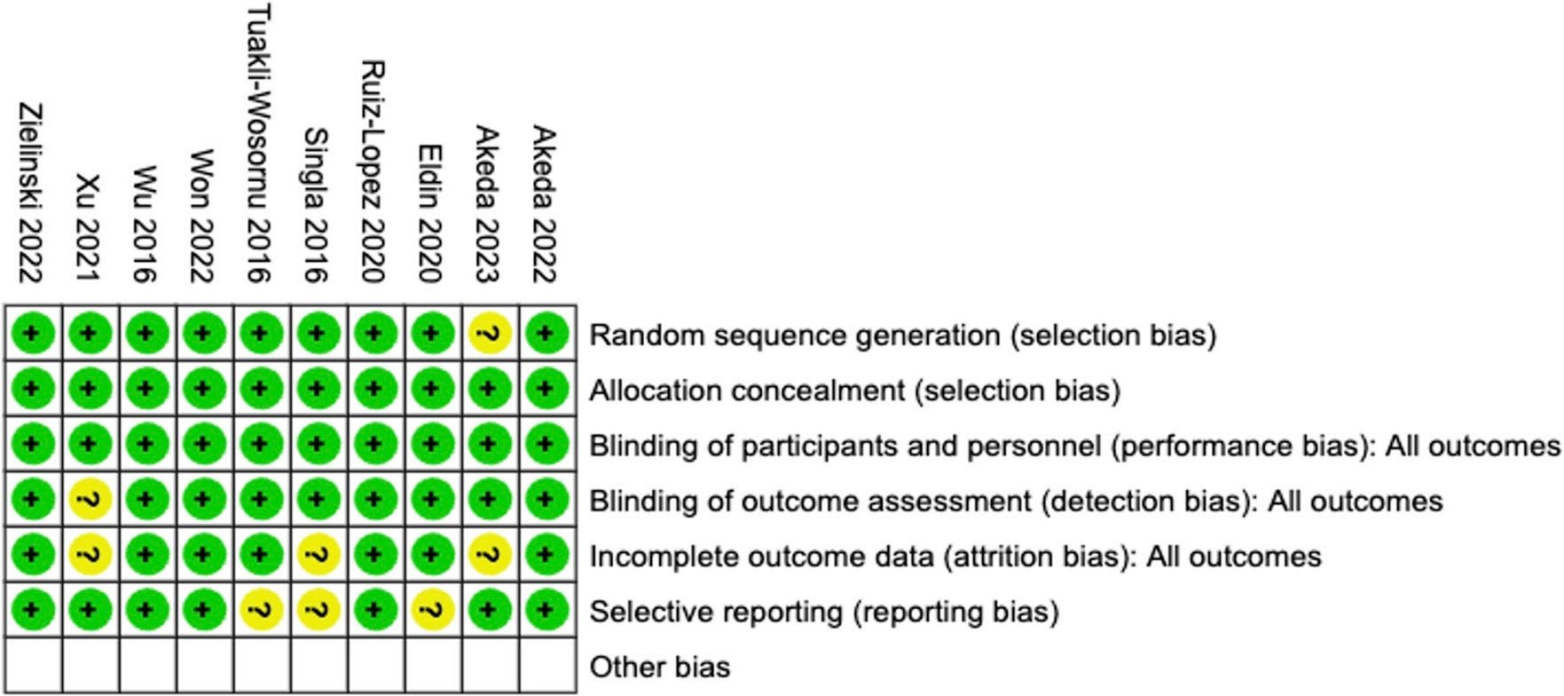
Summary of the Risk of Bias (RoB) assessments using Cochrane's version 2 tool across the ten studies included in the analysis

Cochrane RoB v2.0 (see Fig. 2) indicates strong internal validity throughout the included studies, rendering them reliable and trustworthy. Additionally, examination of the inverted funnel plot (see Supplementary Figs. 1–6) demonstrated a qualitatively symmetrical shape, implying an absence of potential publication bias.

## Discussion

### Study Rationale

In patients with discogenic low back pain, preliminary clinical trials have shown promising results with intradiscal injection of autologous PRP [12]. This approach involves injecting PRP directly into intervertebral discs to promote tissue regeneration and repair. The safety, efficacy and effectiveness of this treatment option have been demonstrated, providing a potential alternative for patients with discogenic low back pain [12]. In addition to intradiscal injection, PRP has also been used in the management of cervico-discogenic pain. A case report and technical note described the use of point of care ultrasound guided cervical intervertebral disc injection of PRP to alleviate pain in a patient with cervical discogenic pain [13•]. This technique involved injecting PRP mixed with lidocaine into the affected disc under ultrasound guidance. The use of PRP in this case resulted in pain relief and improved functional outcomes.

It is important to note that safety of intradiscal PRP injections should be carefully considered. An *in vitro* study investigated safety of intradiscal PRP by examining its antimicrobial properties against Cutibacterium acnes, a bacterium commonly associated with intervertebral disc degeneration. The study found that PRP had antimicrobial effects against C. acnes, suggesting that it may have a protective role in preventing infection following intradiscal injections [14].

The outcomes of our meta-analysis suggest that, in the assessment of vertebrogenic and discogenic pain utilizing VAS scores, PRP treatment did not demonstrate a noteworthy advantage over the control group at the designated time points. Likewise, the analysis employing ODI scores indicated a potential inclination towards improvement in PRP cohort in relating to the control group in the initial month, albeit without reaching statistical significance. Furthermore, no statistically significant distinctions were identified in ODI scores during the subsequent third and sixth-month assessments.

### Supporting and Conflicting Investigations

The use of PRP in vertebrogenic and discogenic pain has been the subject of several studies. Akeda et al., conducted a preliminary clinical trial to determine the safety and efficacy of intradiscal injection of autologous PRP releasate in individual experiencing discogenic low back pain [12]. They found that PRP, which contains autologous growth factors and cytokines, has been extensively used for tissue repair and regeneration in the clinical settings. This suggests PRP may have potential benefits in treating discogenic pain. While some studies support the use of intradiscal PRP for discogenic pain, others have not found significant structural or functional improvement from the included studies [15•, 16•].

Sevgili and Sari observed remarkable pain relief and improved return to pre-illness activity levels with single-level intradiscal PRP injections for discogenic pain [17]. However, Akeda et al. underscored the necessity for larger-scale studies to validate the clinical evidence for PRP in treating discogenic lower back pain [16•]. This need for further research is echoed by Urits et al., who explored radiofrequency ablation of the basivertebral nerve as a potential non-surgical treatment option for vertebrogenic low back pain [18]. Mohammed and Yu highlighted PRP therapy's potential in managing chronic discogenic low back pain but emphasized the imperative for additional evidence from clinical trials [19].

### Implementation of PRP in Patients Suffering Vertebrogenic and Discogenic Pain

Several studies have investigated clinical implementation of PRP in patients with vertebrogenic and discogenic pain [20]. Akeda et al. conducted a critical review of the use of PRP in chronic low back pain and reported that PRP injections have shown promising results in managing this condition [21]. In a preliminary clinical trial, Akeda et al. also found that intradiscal injection of autologous PRP releasate was safe and showed initial efficacy in individuals with discogenic low back pain [12]. Similarly, Lutz et al. assessed the clinical outcomes of higher-concentration PRP injections in patients with chronic lumbar discogenic pain and found that PRP injections led to significant improvements in pain and function [22•]. The use of PRP in the management of discogenic pain has also been supported by studies evaluating its long-term effects [23•]. Akeda et al. conducted a long-term follow-up survey and found that PRP therapy provided sustained pain relief in patients with discogenic low back pain [7•]. Jain et al. conducted a prospective clinical trial and concluded that intradiscal PRP injection was effective in treating discogenic low back pain, especially when higher platelet counts were used [24]. Sevgili and Sari investigated the role of single level intradiscal autologous PRP injection in the treatment of discogenic pain and reported remarkable pain relief and improved activity levels in patients [17].

### Prospects for Future Research

One area of future research might focus on the mechanism of action of PRP in treating vertebrogenic and discogenic pain. Laboratory investigations might consider analysis of the portion of injected substance retained within the degenerative tendon or disc, offering insights into the precise biological pathways responsible for alleviating pain and promoting tissue regeneration [25]. Another avenue for future research is the identification of patient phenotypes that are most likely to respond to PRP treatment. Evaluating patients through clinical examination, considering their inflammatory phenotype, and identifying pain sources and structural changes through imaging could assist in determining those who would derive the greatest benefits from PRP therapy [26•]. Additionally, future studies should aim to establish the long-term effects of PRP treatment in patients with vertebrogenic and discogenic pain. Long-term follow-up surveys and prospective clinical trials can provide valuable insights into the sustained pain relief and functional improvements achieved with PRP therapy.

## Limitations of the Study

We encountered several limitations in the present investigation, including a relatively small sample size, a brief follow-up period, potential patient heterogeneity, and limited generalizability. The modest sample size may curtail the study ability to generalize its findings effectively and attain robust statistical power. The short follow-up period restricts the examination of PRP treatment long-term efficacy, a crucial aspect of chronic pain management. Patient heterogeneity poses a challenge, given the potential variations in the underlying causes and presentations of vertebrogenic and discogenic pain among participants. Furthermore, the studies outcomes may not readily apply to a broader patient population or diverse clinical settings, emphasizing the need for cautious extrapolation of its findings.

### Supplementary Information

Below is the link to the electronic supplementary material.

Supplementary file1 (JPG 442 KB)

Supplementary file2 (JPG 457 KB)

Supplementary file3 (JPG 479 KB)

Supplementary file4 (JPG 398 KB)

Supplementary file5 (JPG 390 KB)

Supplementary file6 (JPG 364 KB)

## Data Availability

No datasets were generated or analysed during the current study.
