# Correction to: The Role of Platelet Rich Plasma in Vertebrogenic and Discogenic Pain: A Systematic Review and Meta-Analysis

**DOI:** 10.1007/s11916-024-01300-z

**Published:** 2024-07-10

**Authors:** Saurabh Kataria, Jeremiah Hilkiah Wijaya, Utsav Patel, Kevin Yabut, Tawfiq Turjman, Muhammad Abubakar Ayub, Nihar Upadhyay, Moinulhaq Makrani, Hisham Turjman, Ahmed Mostafa Abdalla Mohamed, Alan D. Kaye

**Affiliations:** 1 Department of Neurology, Louisiana State University Health Sciences Center at Shreveport, 1501 Kings Highway, Shreveport, LA 71103 USA; 2 Department of Neurosurgery, Siloam Hospital Lippo Village, Tangerang, Banten Indonesia; 3 Mayo Clinic, Jacksonville, FL 32224 USA; 4 Louisiana State University Health Sciences Center, Shreveport, LA 71103 USA; 5 School of Medicine, Royal College of Surgeons in Ireland, Baharain, USA; 6 Department of Internal Medicine, GMERS Medical College, Gotri, Vadodara, Gujarat 390021 India; 7 Dept. of Pharmacology, Parul Institute of Medical Science and Research, Waghodiya, Gujarat 291760 India; 8 Department of Anaesthesiology and Interventional Pain, Louisiana State University Health Sciences Center at Shreveport, Shreveport, LA 71103 USA; 9 Louisiana Addiction Research Center, Shreveport, LA 71103 USA

**Correction to: Current Pain and Headache Reports**

10.1007/s11916-024-01274-y

The article “The Role of Platelet Rich Plasma in Vertebrogenic and Discogenic Pain: A Systematic Review and Meta-Analysis”, was originally published electronically on the publisher’s internet portal on 8 June 2024 with error in ninth author’s family name. ‘Trujman’ should be corrected to ‘Turjman’.

The original article has been corrected.

