# Supplementary figures and images for: The Role of Platelet Rich Plasma in Vertebrogenic and Discogenic Pain: A Systematic Review and Meta-Analysis
Source: Curr Pain Headache Rep. 2024 Jun 8;28(8):825–33. doi: 10.1007/s11916-024-01274-y (PMC11272713; doi:10.1007/s11916-024-01274-y)

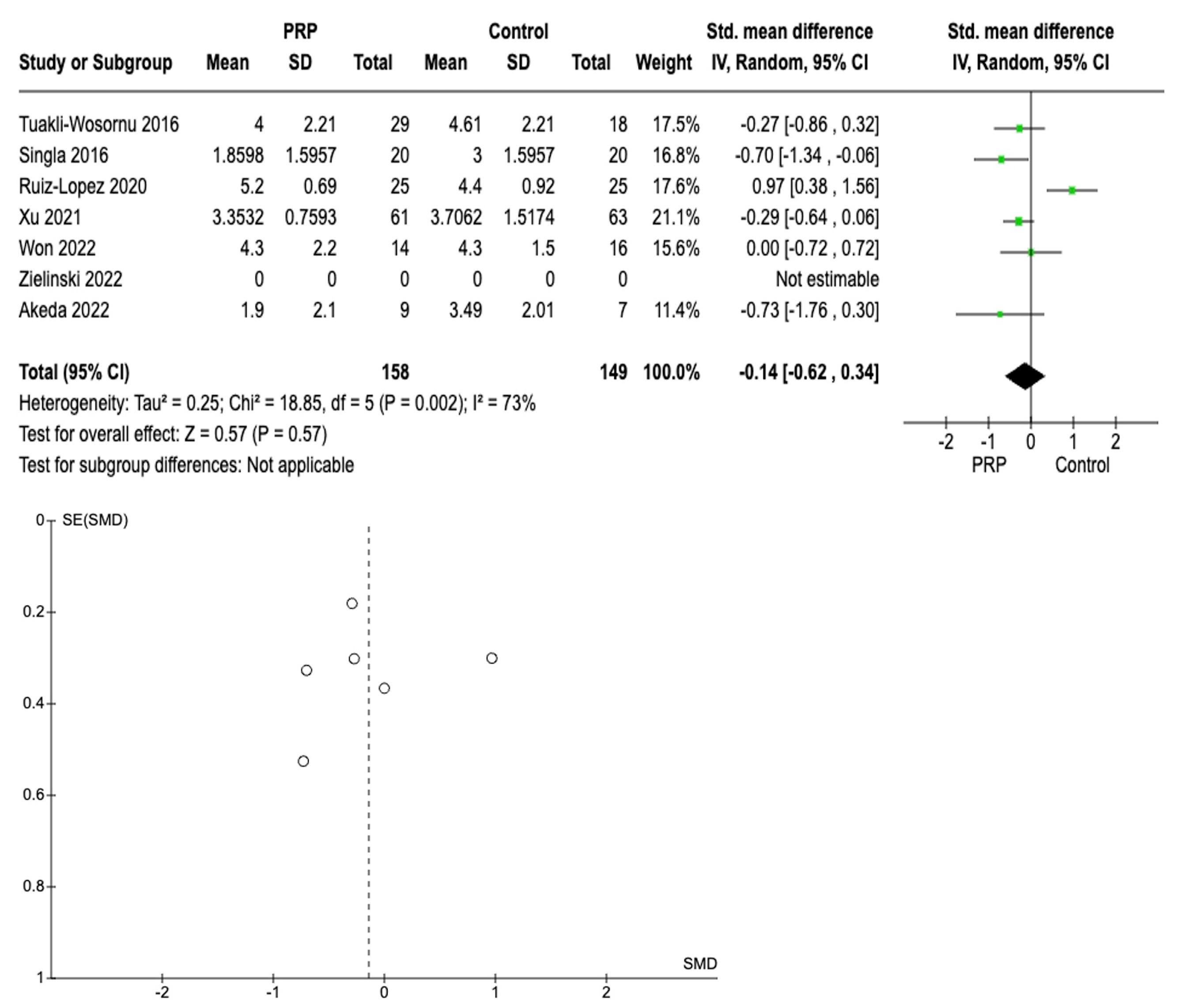

Supplement: Supplementary file 1 — Supplementary file1 (JPG 442 KB) [file 11916_2024_1274_MOESM1_ESM.jpg]

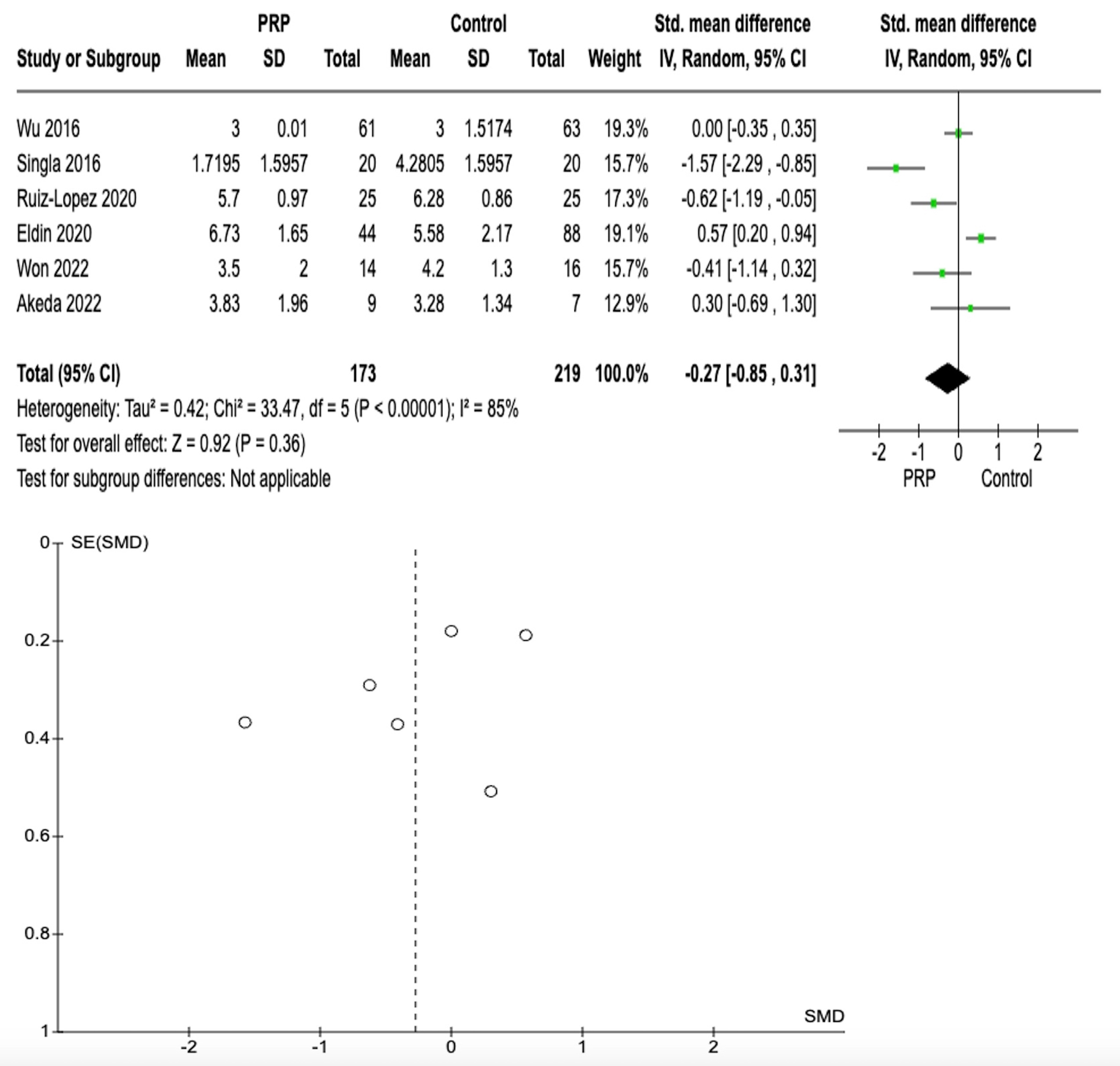

Supplement: Supplementary file 2 — Supplementary file2 (JPG 457 KB) [file 11916_2024_1274_MOESM2_ESM.jpg]

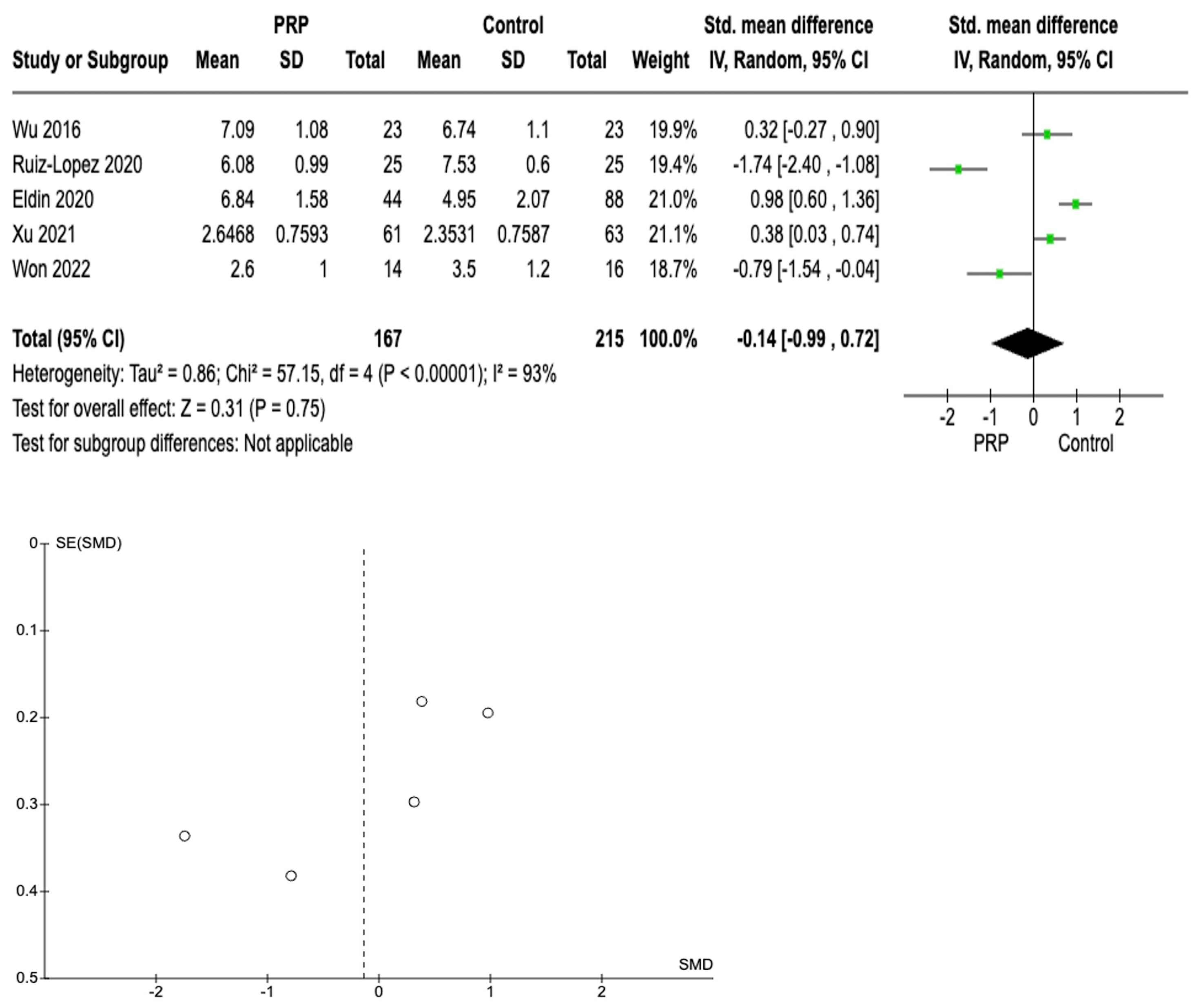

Supplement: Supplementary file 3 — Supplementary file3 (JPG 479 KB) [file 11916_2024_1274_MOESM3_ESM.jpg]

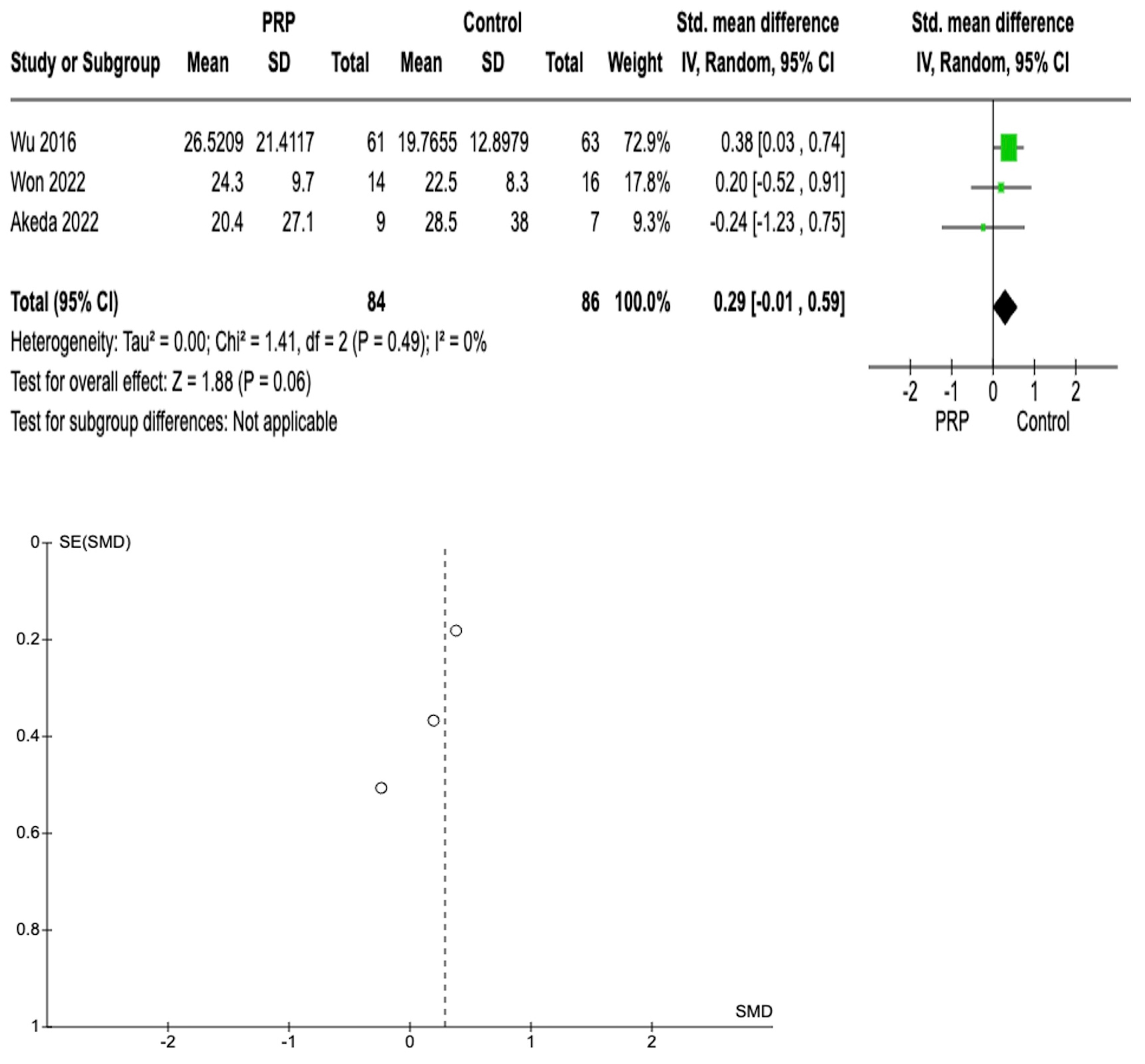

Supplement: Supplementary file 4 — Supplementary file4 (JPG 398 KB) [file 11916_2024_1274_MOESM4_ESM.jpg]

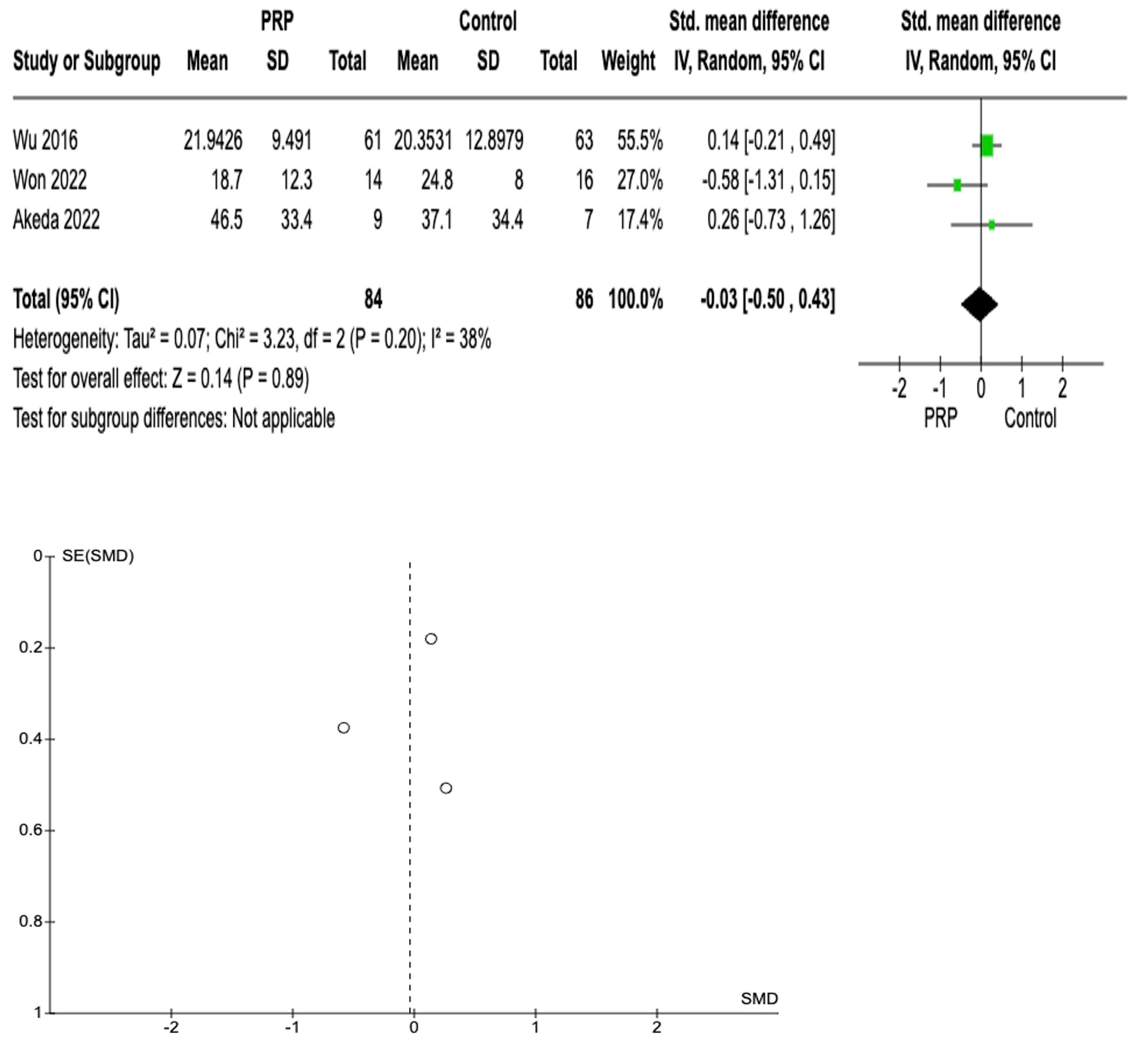

Supplement: Supplementary file 5 — Supplementary file5 (JPG 390 KB) [file 11916_2024_1274_MOESM5_ESM.jpg]

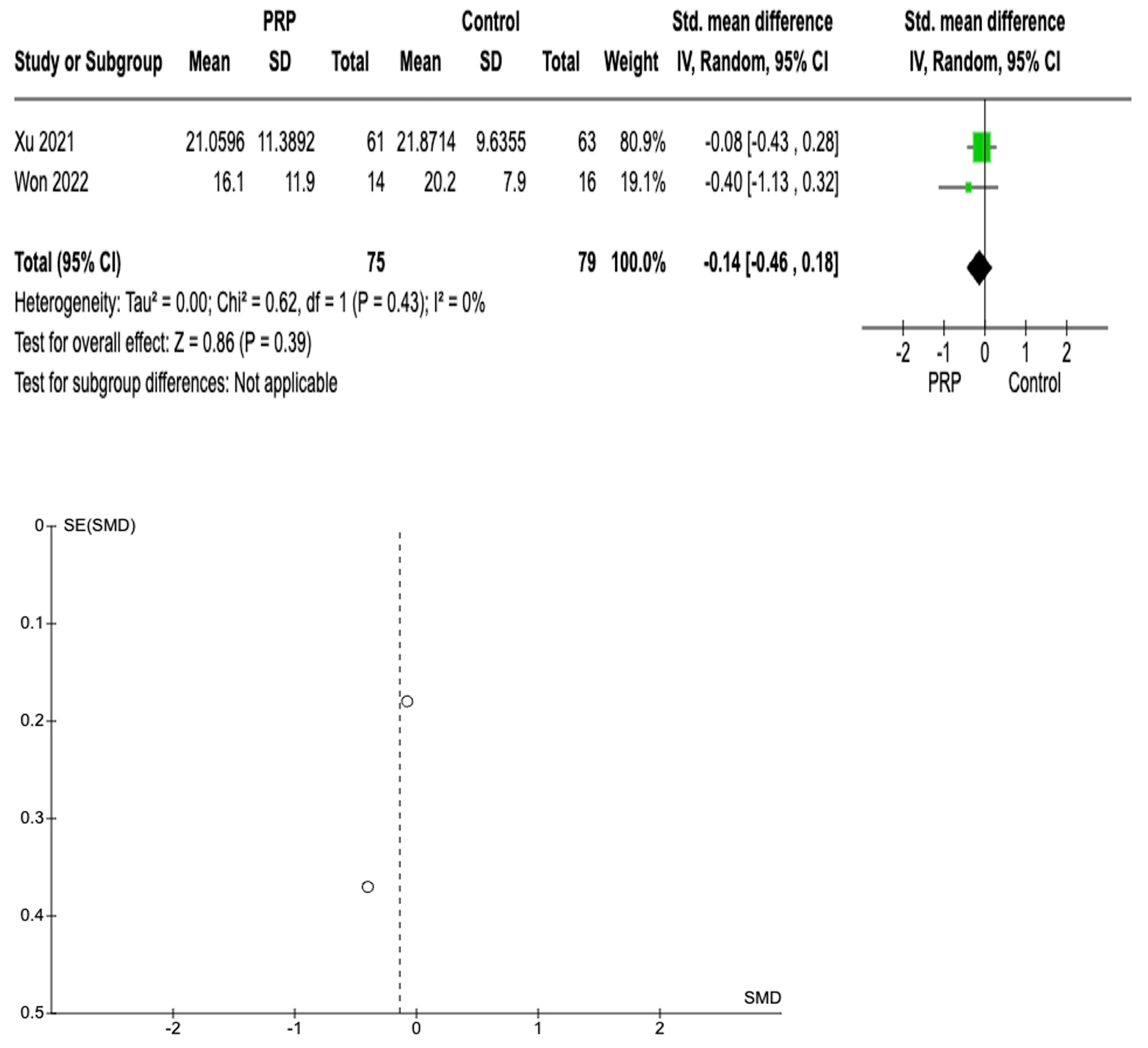

Supplement: Supplementary file 6 — Supplementary file6 (JPG 364 KB) [file 11916_2024_1274_MOESM6_ESM.jpg]
